# Coagulase-negative staphylococci (CoNS) as a significant etiological factor of laryngological infections: a review

**DOI:** 10.1186/s12941-020-00367-x

**Published:** 2020-06-04

**Authors:** Michał Michalik, Alfred Samet, Adrianna Podbielska-Kubera, Vincenzo Savini, Jacek Międzobrodzki, Maja Kosecka-Strojek

**Affiliations:** 1 MML Medical Centre, Warsaw, Poland; 2 Clinical Microbiology and Virology, Spirito Santo Hospital, Pescara, PE Italy; 3 Department of Microbiology, Faculty of Biochemistry, Biophysics and Biotechnology, Jagiellonian University, Kraków, Poland

**Keywords:** Coagulase-negative staphylococci, CoNS, Infections, Laryngology, *Staphylococcus*

## Abstract

This review article shows that coagulase-negative staphylococci (CoNS) are widely responsible for laryngological diseases. General characteristics of CoNS infections are shown in the introduction, and the pathogenicity in terms of virulence determinants, biofilm formation and genetic regulation mechanisms of these bacteria is presented in the first part of the paper to better display the virulence potential of staphylococci. The PubMed search keywords were as follows: CoNS and: nares infections, nasal polyps, rhinosinusitis, necrosing sinusitis, periprosthetic joint infection, pharyngitis, osteomyelitis of skull and neck bones, tonsillitis and recurrent tonsillitis. A list of laryngological infections and those related to skull and neck bones was presented with descriptions of the following diseases: rhinosinusitis, necrotizing sinusitis, nasal polyps, nares and nasal skin infections, periprosthetic joint infections, osteomyelitis, pharyngitis, and tonsillitis. Species identification and diagnostic problems challenging for diagnosticians are presented. Concluding remarks regarding the presence of CoNS in humans and their distribution, particularly under the effect of facilitating factors, are mentioned.

## General characteristics of coagulase-negative staphylococci (CoNS) infections

The significance of CoNS in infectious medicine became apparent in the late 1970s following a series of articles on the isolation of these bacteria from diagnostically documented infections in humans, as shown by several authors [1–6]. The definition of CoNS as a heterogeneous group of staphylococci is based on diagnostic procedures that fulfil the clinical need to differentiate between *Staphylococcus aureus*, commonly known as strongly pathogenic, and other staphylococci classified as non- or less pathogenic [7]. CoNS are characterized by fewer virulence factors than *S. aureus*, especially factors responsible for aggression, so they are considered less pathogenic. However, the virulence of both CoNS and coagulase-positive staphylococci (CoPS), represented by *S. aureus*, were compared using various animal models of experimental infections [8–13]. The authors showed that some CoNS species demonstrated skin lesions similar to the lesions generated by *S. aureus* [11]. Generally, the pathogenic potential of CoNS could be accepted when both biochemical and genetic molecular methods were introduced to analyze the pathogenesis and mechanisms of infections [7, 14]. In the last two decades, the medical importance of this group of bacteria has increased, as reported by clinicians and microbiologists [15, 16]. There is evidence that CoNS are responsible for a variety of infections that differ in localization, manifestation or course of infection. However, staphylococcal bacteria are opportunistic pathogens that are present in the skin and mucous membranes of healthy individuals and become true pathogens mostly for predisposed patients, i.e., immunocompromised individuals, patients with catheters, prosthetic implants, dialysis, and oncological diseases, and neonates [17–20].

As pathogens, CoNS are involved in a broad range of diseases in deep organs, including bones, the central nervous system, the heart or joints [21]. However, CoNS are typical opportunistic bacteria that not only colonize healthy individuals but also represent one of the major hospital pathogens with a substantial increasing impact on human life and health [7]. The presence of CoNS in the skin and mucous membranes of the host is a main source of endogenous infections [7, 22]. In addition, bacteria are transmitted among diverse hosts by crossing species barriers and during medical procedures, especially invasive ones [23, 24]. Regarding otorhinolaryngology, CoNS are always present in the skin and mucous membranes of the nose, respiratory tract, oral cavity and alimentary canal [25, 26]. In the field of laryngology, the main papers on CoNS pathogenicity focus on diseases such as bacteremia, septicemia, pneumoniae, endocarditis or urinary tract infections, but there are few reports on laryngological diseases such as rhinosinusitis and sinusitis, both acute and chronic, and infections of the frontal sinus, throat, larynx, nares, polyps, tonsils, and trachea. A dozen of the over 50 described CoNS species have been commonly reported in these diseases, including *Staphylococcus epidermidis, Staphylococcus haemolyticus, Staphylococcus lugdunensis, Staphylococcus warneri, Staphylococcus xylosus, Staphylococcus hominis, Staphylococcus capitis, Staphylococcus simulans, Staphylococcus sciuri, Staphylococcus cohnii, Staphylococcus lentus,* and *Staphylococcus chromogenes*, although other rare species, such as *Staphylococcus pettenkoferi*, have been documented [7, 27, 28].

## Pathogenicity: virulence determinants, biofilm formation and genetic regulation of CoNS species

CoNS possess numerous diverse strategies to both cause infection and survive in the host. In comparison to *S. aureus*, this group of bacteria exhibits lower pathogenic potential, but less is known about CoNS virulence mechanisms. Recent studies have focused on the ability of CoNS to produce a variety of extracellular enzymes, such as proteases, elastases, esterases, lipases, and phospholipases [29–32], as well as the production of toxins, such as families of hemolysins, enterotoxins, exfoliative toxins, and even TSST-1 (Toxic shock syndrome toxin-1) [7, 33–36]. The enzymes and toxins presented play crucial roles in some of the effects of staphylococci on host organisms, including virulence factors in tissue destruction and additionally as spreading factors facilitating invasion into nearby tissues. In general, CoNS species lack the virulence determinants responsible for aggression, but they possess the ability to adhere, invade and persist [14, 31]. Furthermore, a critical CoNS property is the ability to colonize the surfaces of medical devices by the formation of a three-dimensional structured matrix composed of bacteria and extracellular biopolymers-biofilm. Increasing evidence suggests that biofilms may form on abiotic surfaces of medical devices or on biotic surfaces, such as host factor-coated foreign material or host tissue [37, 38]. Biofilm-associated infections are extremely difficult to eradicate because within the biofilm, bacteria are protected against the immune system of the patient and antibiotic therapy [39, 40]. Identification of the factors that increase CoNS pathogenicity is a great need, especially for clinicians and microbiologists. Studies described to date have demonstrated that *S. epidermidis* isolates differ regarding antibiotic resistance gene acquisition (e.g., SCC*mec*), biofilm forming capacities (e.g., *ica* locus), metabolism (e.g., arginine catabolic mobile element ACME) and the presence of insertion sequence (IS) elements (e.g., IS256) [41–44]. It is very important to extend such studies to a larger number of isolates and species, but there is strong evidence that horizontal gene transfer is responsible for virulence factor transmission between species [45–48]. Staphylococcal pathogenesis is a process involving an array of extracellular proteins and biofilm and cell wall components that are coordinately expressed in different phases of infection. The expression or suppression of two divergent loci, accessory gene regulator (*agr*) and staphylococcal accessory regulator (*sar*), are recognized as key regulators of virulence in staphylococcal infections [49, 50]. The *agr* system is known to modulate virulence factors such as nucleases, proteases, lipases and the expression of surface binding proteins, and *sar* can modulate both the *agr* system and independently form an *agr* locus of cell wall-associated proteins such as fibronectin-binding protein, adhesins, protein-A and exo-proteins. When the bacterial population is low, the expression of adherence proteins is triggered for attachment to host tissue and toxins are produced once the infection is established [51].

## General descriptions of laryngological infections

Laryngology, otolaryngology, is generally a broad medical discipline that covers the diagnosis, therapy, and prophylaxis of diseases of the upper respiratory tract and additional organs present in the neck and head, including throat, larynx, nose, ears, and other organs in these parts of body [52]. Recently, important medical questions in laryngology have been raised regarding oncological and infectious diseases [53, 54]. Statistically viral and bacterial infections of the respiratory tract that spread into other anatomical niches are the most identified in epidemiological analysis. Staphylococci, particularly CoNS, can cause local infections, but they are also responsible for pathological complications after expanding from laryngological areas to other areas, including skin, and heart, and even sepsis in newborns as well as nosocomial infections [55–61]. Moreover, an increasing number of fungal infections has recently been reported in laryngology studies, and these infections are related to the overconsumption of antibiotics and patients with immune disorders.

## Rhinosinusitis

The rhinosinus is the space in the front part of the facial skeleton that is filled with air and mucous membranes with ciliated epithelium, including mucous glands. In addition, inflammation proceeds synchronously because of the anatomical and physiological relations of the mucous membranes of the nose and rhinosinus, which is the reason for the Latin name used for inflammation of mucous membranes of nose and rhinosinus–rhinosinusitis. Acute rhinosinusitis (ARS) appears the most frequently in the course of viral cold disease, whereas chronic rhinosinusitis (CRS) is a common source of CoNS isolation [62]. CRS represents a heterogeneous group of diseases resulting from the multifaceted interactions between the individual patient and environment. Chronic rhinosinusitis is one of the most common chronic disorders, present in up to 28% of European or American populations [63]. The mechanisms of this disease are still under careful molecular analysis. The main underlying causes of rhinosinusitis are various bacteria, but the most commonly isolated are gram-positive cocci. The predominant organisms identified are streptococci and staphylococci. Bacterial infection plays an important role in CRS as either a causative or exacerbating factor [64, 65]. However, the role of CoNS in CRS remains “controversial” [66]. For a significant subpopulation of patients, only CoNS were isolated, while another population had other positive cultures, and a small group had no bacterial growth [66].

Microbiological analysis of samples from CRS patients revealed that mainly CoNS were identified, followed by *S. aureus* and gram-negative rods, including *Pseudomonas aeruginosa*, *Stenotrophomonas maltophilia*, *Escherichia coli*, and *Serratia marcescens* [65]. The most frequently found CoNS species in CRS patients was *S. epidermidis*, and other CoNS, such as *S. lugdunensis*, *S. capitis*, *Staphylococcus saprophyticus*, *S. haemolyticus*, and *Staphylococcus saccharolyticus* and to a lesser degree other minor CoNS, were present in 87% of cases [63, 67].

Molecular genetic studies have shown that the pathologic ability of CoNS such as *S. epidermidis* depends on genes associated with biofilm formation that have only been found in certain strains [68]. However, knowledge of the importance of bacteria and microbial biofilms in the etiology of CRS is still incomplete. Some authors have determined the association between positive CoNS culture at the time of functional endoscopic sinus surgery (FESS) and CRS severity. Simultaneously, bacteria other than CoNS have been isolated: *Streptococcus pneumoniae* (30–70%), *Streptococcus pyogenes* (3–7%), *Streptococcus* spp., *Moraxella catarrhalis* (12–28%), *Enterobacter* spp., *Cutibacterium acnes* [66], *S. aureus* (27%), *Haemophilus influenzae* (27%), *P. aeruginosa* (22%), *Peptostreptococcus* spp. (20%), *Bacteroides* spp. (19%), *Klebsiella pneumoniae* (11%), and *E.coli* (11%) [69]. Other authors analyzed the exacerbation of CRS in 125 patients and identified CoNS as the most common organism, followed by *S. aureus* [70]. Studies on cultures from 394 CRS patients revealed that aerobes were predominant, and CoNS were common (24%), followed by *S. aureus* (19%) and *Streptococcus viridans* (10%) [71]. Despite the high prevalence of CoNS and their controversial effect on CRS, some reports have shown that the bacteria as the sole positive culture result did not result in increased CRS disease burden [66]. The presence of CoNS alone was independently associated with no history of prior FESS. However, some authors showed no significant difference in CRS disease severity between patients with CoNS as the sole positive culture result and no bacterial growth, but phenomena such as horizontal virulence gene transfer, quorum sensing, and mechanisms of gene expression/suppression necessitate further studies on a larger number of patients and isolates to evaluate whether the effect of CoNS differs in various subgroups [66, 69]. The role of CoNS in CRS pathogenesis is not completely understood because the bacteria that colonize the nasal cavity under physiological conditions have been interpreted as contamination in microbiological analysis by few authors. Most authors have reported an important role for CoNS in CRS pathogenesis and the mechanism of the disease because of the extracellular production of virulence factors such enzymes and toxins, biofilm formation, and virulence or resistance gene transfer among the bacteria colonizing these anatomical areas. However, there are few factors that may affect the detection of CoNS as etiological factors in such infections. The frequency of CoNS isolation at the infection site and the molecular typing of CoNS to determine the clonality of the isolates is crucial in determining the real etiological factor. Moreover, the presence of invasive medical devices and the immune status of patients should be considered, as immunocompromised patients are more susceptible to CoNS infection.

## Necrotizing sinusitis

Necrosing sinonasal infection often involves infectious agents such as viruses, bacteria, fungi, and parasites [72–74]. However, the only reported CoNS species responsible for acute necrotizing sinusitis is *S. lugdunensis,* which was reported in hospitalized patients with metastatic prostate adenocarcinoma [75]. Additionally, severe acute necrotizing sinusitis was complicated by periorbital cellulitis and ulceration from the maxillary sinus to the hard palate. The only pathogen to be identified in three independent biopsy samples was *S. lugdunensis* [75]. The species identification of organisms was verified by the Health Protection Agency Respiratory and Systematic Infection Reference Laboratory using advanced methodology. Interestingly, *S. lugdunensis*, in addition to being a skin commensal of the limbs, groin and nose vestibule of healthy adults, has also been isolated from acute oral infections [76], prosthetic joints, endovascular, skin and soft tissue infections; in individual cases, *S. lugdunenesis* has been isolated as an agent of osteomyelitis, which can lead to laryngological implications in terms of significant effects on the head or face bones. Moreover, *S. lugdunensis* is a major pathogen for heart valves, both native and prosthetic, as it causes very aggressive endocarditis [75, 77].

## Nasal polyps

Many staphylococci colonize the human nose vestibule; however, the main question relates to *S. aureus*, as it is unknown whether and how this bacterium adapts to this particular ecological niche during colonization. Staphylococci are among the bacterial species associated with increasing invasive diseases and drug resistance distribution in humans [78]. The analysis of the composition of the human nasal culturome revealed that *S. epidermidis* colonizes almost all individuals (97%), followed by *S. haemolyticus* (44%), *S. hominis* and *S. capitis* (each 41%), *S. warneri* (32%) and *S. lugdunensis* (26%) [79]. The basis of colonization with strains carrying virulence determinants and/or resistance results from fibronectin-, fibrinogen-, and collagen-binding surface proteins responsible for adhesion and further infection [80]. Polyps are abnormal growth tissue complexes that originate from mucous membranes and are generally present in any organ. In laryngology, polyps are interpreted as disorders or lesions of an inflammatory nature or caused by injury; most cases involve a history of trauma [81]. Therefore, polyps occur elsewhere in the body where there is a mucous membrane. The presence of polyps and accompanying mucosal edema is one of the main observations at examination [82]. The polyps may be involved in chronic sinusitis. CRS with polyps can be present not as a single disease entity but as nasal symptoms of many different diseases [83]. Environmental microorganisms affect the host and cause symptoms in CRS. Bacteria involved in the disease play an important role, and bacterial biofilms present in CRS patients are responsible for noneffective therapy even after surgical and antibiotic interventions [63]. A group of patients with nasal polyps may have different disease etiologies, and it is well documented that culture results are difficult in post-ESS patients. CRS patients with CoNS as the sole positive culture result were significantly more likely to have nasal polyps [66]. Analysis of the bacteriological profile of the patients showed that CoNS, *S. aureus*, *Streptococcus* sp., *Haemophilus* sp., *Enterobacter* sp., and *Corynebacterium* sp. appear to be more frequently associated with patients with chronic rhinosinusitis (CRS) with nasal polyps than with patients with CRS without nasal polyps or control individuals, where the most common aerobic bacteria were CoNS, *Corynebacterium* spp., *S. aureus*, and *H. influenzae*. Additionally, few anaerobic bacteria and fungi have been isolated from various study groups [84]. Authors of another project reported no significant differences in isolation rates among three groups of CRS patients, i.e., with polyps, without polyps, and the control group, although the two most common bacterial species were CoNS and *Corynebacterium* spp. in CRS with polyps group; CoNS, *Corynebacterium* spp., and *S. aureus* in CRS without polyps group; and CoNS, and *S. aureus* in the control group. Finally, CoNS were the most common species among the three groups [85].

## Nares and nasal skin infections

The human nares and skin flora are normally largely composed of different species of CoNS and coryneforms, as reported by many authors [1, 2, 86–88]. Among all aerobic bacteria, CoNS are the group of organisms with the best characteristics for growth and multiplication in cutaneous recesses enriched with sebum and sweat because of the many extracellular enzymes they produce, enabling the use of skin substrates, particularly neutral fatty acids of sebum [89–91]. Infections with *Staphylococcus* bacteria can result in a variety of skin conditions, including cellulitis, furuncles, impetigo or staphylococcal scalded skin syndrome (SSSS). Some CoNS strains, such as *S. epidermidis* and *S. haemolyticus*, share the same habitats as *S. aureus* and permanently or transiently colonize the anterior nares, nose vestibule, and other regions of skin and mucous membranes, acting as a source of infection [92]. Therefore, both *S. aureus* and CoNS are often recovered from the same diagnostic specimen in parallel [93].

CoNS infections of nares or nasal skin have been reported in a minority of cases in comparison to *S. aureus* infections, and they are always combined with immunocompromised or directly post-surgery hospitalized patients [94, 95]. Common types of nasal staphylococcal infections include folliculitis, which is an infection of one or many hair follicles; furuncle, also known as boils, which are deep infections around the hair follicle or oil glands that contain pus; or nasal vestibulitis, an infection of the front area of the nasal cavity that may cause crusting and bleeding. There are various symptoms of nasal staphylococcal infections, such as crusting, swelling, lesions with pus secretion, light bleeding, pain, redness or fever [96]. Therefore, nasal carriage of CoNS, especially methicillin-resistant CoNS, appears to play a key role in the epidemiology and pathogenesis of infections in nares or upper respiratory tracts [94]. Increasing evidence suggests that nasal CoNS are also reservoirs for mupirocin resistance, which may therefore be transmitted to *S. aureus*. Mupirocin is the first-line drug treatment to eradicate *S. aureus* nasal colonization, which may fail, although rarely, due to the transfer of underlying genetic elements from CoNS to *S. aureus* [97, 98]. Over the last few years, CoNS have become important as causative agents of hospital-acquired bacteremia and surgical site infections. Thus, CoNS colonizing anterior nares and skin are severe pathogens responsible for infections and are additionally often associated with multiple antimicrobial-resistance mechanisms, including antibiotic resistance of various other pathogens [51, 52].

## Periprosthetic joint infections

In laryngology, temporomandibular joint (TMJ) infections are still a challenge, because there is no single leading disease [99]. The TMJ joins the mandible with the skull, and pain can mimic infections of the inner ear. The TMJ is part of a larger system known as the stomatological system or locomotor system of the masticatory apparatus. This is a very complex system under permanent and dynamic changes throughout life. Infections of the TMJ in both intracapsular and pericapsular courses are rare diseases [100]. The predisposing factors for contributing to TMJ infections are divided into local factors, such as blunt trauma, history of joint diseases and burn, and systemic factors including autoimmune disease and overconsumption of medicines with special reference to steroids [57, 101, 102]. Therefore, there are various reasons for TMJ infections; however, it is difficult to indicate the most important. Some authors have shown fatigue of TMJ muscles, orthodontic disorders, trauma of the head or spine, and stress as reasons for TMJ infections [103]. Analysis of ankylosis and arthritis showed that the most common causes of the disease are trauma and infection [104], and psychological aspects have also been reported as factors in nonorganic TMJ dysfunction [105]. The invasion of bacteria into the joint space can occur through several routes, but hematogenous spread indicates that blood circulation is the most common route. The other methods are adjacent, contiguous infected tissues and direct centesis into the joint cavity by direct inoculation [100]. Among these, the most prevalent route is hematogenous spread originating from primary infection sites [106]. The typical bacteria causing TMJ infections are *Streptococcus* spp., *S. aureus*, *S. epidermidis*, *Neisseria gonorrhoeae*, *H. influenzae*, and *P. aeruginosa* [107, 108]. The tests used to confirm species identifications showed that CoNS, such as *S. lugdunensis*, *S. epidermidis*, *S. haemolyticus*, *S. xylosus, S. warneri*, and *S. hominis*, were isolated from fluid secreted by the temporomandibular joint of patients with muscle pain [109]. Additionally, *S. capitis*, a multidrug-resistant strain with documented potential for both human disease and nosocomial spread, was isolated from a group of patients [110]. An accompanying chronic orofacial muscle pain is associated with membrane-damaging toxins from CoNS (MDT-CoNS) or *S. aureus*. Previous reports suggest that membrane-damaging toxins produced by CoNS are associated with the development of chronic disease [111, 112]. Additionally, an evaluation in the distribution of *Staphylococcus* spp. in different phases of prosthetic joint infection (PJI) was observed, and the predominant pathogens were CoNS followed by *S. aureus*. Almost equal proportions of CoNS and *S. aureus* were observed in the delayed phase. CoNS were the predominantly identified organisms in the early phase, whereas *S. aureus* was observed primarily in the late phase [113]. In many medical disciplines, such as infectious laryngology, orthopedic infections due to CoNS presenting antibiotic-resistant profiles are increasing. Soft tissues and bone implant-associated infections are caused by CoNS, with the most prevalent species being *S. epidermidis*. These infections are considered difficult to treat because of the ability of the bacteria to grow in biofilms and form small-colony variants, including persistent organisms [114, 115].

## Osteomyelitis

The discipline of laryngology also concerns diseases of the skull and neck bones. Osteomyelitis generally refers to laryngology-related inflammatory diseases of bones caused by microorganisms and leads to bone destruction. The most frequent microorganisms isolated from osteomyelitis are *S. aureus* and CoNS, with a predominance of *S. epidermidis* [116]. However, a new CoNS species, *S. pettenkoferi*, has been described as causing human osteomyelitis [117]. The disease otitis or otitis externa can also cause complications such as skull base osteomyelitis (SBO) [118]. In particular, orthopedic devices are used for bone fixation or joint replacement and are receptive/susceptible to commensal bacteria, mainly CoNS. The therapy of such infections is expensive, and the infections result in high patient morbidity [119]. Bone infections caused by CoNS are associated with a high prevalence of methicillin resistance, and broad spectrum oral antibiosis was demonstrated in a predominantly diabetic population [116]. In advanced laryngology concerning surgical reconstructions of bones and/or accompanying soft tissues, infections due to CoNS and methicillin-resistant strains are increasing [114]. CoNS are often responsible for cases of chronic osteomyelitis and otitis, especially in patients with orthopedic prostheses or implants. Chronic courses of osteomyelitis or ostitis were caused by the following CoNS species: *S. epidermidis*, *S. haemolyticus*, *S. simulans*, *S. warneri*, *S. sciuri*, *S. cohnii*, *S. hominis*, *S. lentus*, *S. chromogenes*, and *S. pettenkoferi* [120]. Additionally, other chronic forms of osteomyelitis such as suppurative osteomyelitis of the jaws are caused by various bacteria, with a significant portion being CoNS [121].

## Pharyngitis, throat infection

Pharyngitis is a disease of the throat, infection and inflammation caused by viruses, eubacteria, or *Mycoplasma pneumoniae*. The bacteria, that cause these infections include group A/C/G/B streptococci, *Fusobacterium,* and *N. gonorrhoeae*, but *Str. pyogenes* and other streptococci are the predominant species [52]. CoNS are generally considered nonpathogenic, and their presence in clinical material has been interpreted as contamination by normal microbiota present in the mucous membranes of the oral cavity and upper respiratory tracts. However, there are few reports on CoNS in cultures from throat infections, but these studies were mostly in children, and the role of CoNS as causative agents should be further investigated [122–124].

Tonsillitis (T) and recurrent tonsillitis (RT) are caused by viruses, bacteria, *Chlamydia* and fungi. The symptoms of tonsillitis and pharyngitis are similar. The most frequently isolated organisms from T or RT are *S. aureus* strains, from 70% of patients, followed by *Streptococcus* sp. from groups A, B, and C (and G), and the following species dominant as well: beta-hemolytic *Str. pyogenes*, *H. influenzae*, *Haemophilus parainfluenzae*, *Klebsiella pneumoniae*, *Klebsiella oxytoca*, *Moraxella catarrhalis*, *Citrobacter koseri*, *Enterobacter cloacae, Enterobacter aerogenes, E. coli*, and *P. aeruginosa* [125–127]. Aside from streptococci, all other species are considered able to colonize the throat and could be found in recurrent tonsillitis due to gastroesophageal reflux. CoNS have also been isolated in cultures from T and RT infections, but they are reported as accompanying bacteria and culture contaminants [128, 129]. Such results and the involvement of *S. aureus* in the etiology of T and RT have not yet been fully understood because CoNS and occasionally *S. aureus* constantly colonize mucous membranes of the oral cavity and upper respiratory tracts. Furthermore, staphylococcal internalizations in deeper layers or even inside of tonsils, as well as in single cells of patients, have been observed [125, 130].

## Species identification and diagnostics problems

Challenging identification processes may lead to a lack of noted infections caused by staphylococci other than *S. aureus* and their spread in the environment. Commonly used routine diagnostic methods, such as culture-dependent phenotypic tests, including automated systems such as Vitek 2 (bioMérieux, La Balme Les Grottes, France), BD Phoenix (BD Diagnostic Systems, Sparks, MD, USA), and matrix-assisted laser desorption ionization–time of flight mass spectrometry (MALDI-TOF MS), both with 16S rRNA gene sequencing, are usually not precise enough to carefully assign *Staphylococcus* species [28, 131–134]. Many members of the *Staphylococcus* genus are closely phylogenetically related, and the real impact of CoNS species as infectious etiological factors may remain underreported. The implementation of reliable genetic methods in clinical practice will improve the identification process and result in faster and more precise diagnosis of staphylococcal infections. As shown in our review article, in laryngological infections, staphylococci often coexist with other opportunistic and pathogenic bacteria, complicating the identification process. The new genetic diagnostics approach, based on next generation sequencing, may be used for the identification of whole species content in polymicrobial clinical samples. The well-curated and publicly available reference sequence dataset for *Staphylococcus* species will allow the introduction of this approach in all microbiological laboratories with access to NGS (next-generation sequencing) platforms and may be used in diagnosing laryngological infections [28, 135, 136].

## Concluding remarks

CoNS are a heterogeneous group of gram-positive bacteria that colonize human or animal skin and mucous membranes and are distributed from these niches into the environment. Although CoNS are present everywhere, they multiply in humans or animals only. Under physiological conditions, CoNS are typical saprophytes, but under exposure to additional conditions, known as infection-facilitating factors, their character changes from saprophytic to pathogenic. Therefore, CoNS are responsible for various infections of different localizations, manifestations or courses. This review article showed that CoNS are widely present in laryngological diseases. Their presence in clinical materials originating from laryngological patients presents a new challenge for both clinicians and microbiologists; showing these bacteria in new light. Known in the past as “skin staphylococci”, CoNS were interpreted as accompanying bacteria or contamination in diagnostic samples. Today, based on recent reports from advanced microbiological laboratories using molecular diagnostic methods, it is known that CoNS are severe pathogens and require increased infection prevention programs with hygiene discipline in hospitals. Moreover, improved didactic programs are needed to better understand the role of CoNS in laryngological diseases with the primary aim of reducing the number of staphylococcal infections in patients.

## Data Availability

Not applicable.

## Abbreviations

ACME: Arginine catabolic mobile element
*agr*: Accessory gene regulator
ARS: Acute rhinosinusitis
CoNS: Coagulase-negative staphylococci
CoPS: Coagulase-positive staphylococci
CRS: Chronic rhinosinusitis
FESS: Functional endoscopic sinus surgery
IS: Insertion sequence
MDT-CoNS: Membrane-damaging toxins from coagulase-negative staphylococci
NGS: Next-Generation Sequencing
PJI: Prosthetic joint infection
RT: Recurrent tonsillitis
*sar*: Staphylococcal accessory regulator
SBO: Skull base osteomyelitis
SSSS: Staphylococcal scalded skin syndrome
T: Tonsillitis
TMJ: Temporomandibular joint
TSST-1: Toxic shock syndrome toxin-1
